# Severe Vaping Product Use‑Associated Lung Injury in a Healthy Young Adult: A Case Report

**DOI:** 10.7759/cureus.89495

**Published:** 2025-08-06

**Authors:** Nouf A Al Alhadi, Abdulrahman K Alamer, Rawan H Albalawi, Anas E Ahmed

**Affiliations:** 1 Internal Medicine, King Fahad Central Hospital, Jazan, SAU; 2 College of Medicie, King Faisal University, AlAhsa, SAU; 3 Faculty of Medicine, Tabuk University, Tabuk, SAU; 4 Community Medicine, Jazan University, Riyadh, SAU

**Keywords:** acute hypoxemic respiratory failure, corticosteroid therapy, e‑cigarette, ground‑glass opacities, lung injury, nicotine‑free vaping, public health, vaping, vaping product use‑associated lung injury

## Abstract

Vaping product use-associated lung injury is a recently recognized respiratory illness that can occur in users of e-cigarettes or vaping products, including those marketed as nicotine‑free. We describe a previously healthy, 22‑year‑old male, non‑smoker who presented with progressive shortness of breath, non‑productive cough, pleuritic chest pain, low‑grade fever, and fatigue. He reported daily use of a flavored nicotine‑free vaping device for eight months, with increased use in the preceding month, including cartridges purchased from unregulated online sources. Examination revealed hypoxemia, tachypnea, and diffuse inspiratory crackles. Laboratory studies showed leukocytosis and elevated inflammatory markers, while infectious and autoimmune evaluations were negative. High‑resolution computed tomography demonstrated bilateral ground‑glass opacities with dependent predominance and subpleural sparing. A presumptive diagnosis of vaping product use-associated lung injury was made. The patient improved rapidly with supplemental oxygen and corticosteroid therapy, and at the three‑month follow‑up, he had complete symptom resolution, normal pulmonary function, and near‑complete radiographic recovery. This case highlights that severe lung injury can occur in healthy young adults using nicotine‑free vaping products from informal sources, emphasizing the need for thorough vaping histories, careful exclusion of alternative diagnoses, and public health interventions to regulate product safety.

## Introduction

Vaping product use-associated lung injury is an emerging public health concern first recognized in 2019, characterized by acute or subacute respiratory failure in individuals with recent use of e-cigarettes or vaping products [1,2]. While initially identified during an outbreak linked to tetrahydrocannabinol (THC)‑containing cartridges adulterated with vitamin E acetate, subsequent reports have demonstrated that this lung injury can also occur in users of nicotine‑only or purportedly nicotine‑free vaping devices [2-4].

The underlying mechanism is not fully understood but is thought to involve a combination of direct chemical toxicity, thermal injury, and inflammatory responses triggered by inhalation of aerosolized substances and contaminants [2,5]. Clinical presentation varies widely, from mild respiratory symptoms to severe hypoxemia requiring mechanical ventilation, and may include constitutional and gastrointestinal manifestations, making timely diagnosis challenging [2,3].

Imaging typically shows bilateral pulmonary infiltrates with ground‑glass opacities, while microbiological evaluation is negative for infectious causes [1,3]. The diagnosis is primarily one of exclusion, with management focused on supportive care, corticosteroid therapy, and complete cessation of vaping [2-4]. Although cases are reported most frequently among adolescents and young adults, occurrence in non‑smokers without other inhalational exposures underscores the importance of clinician awareness. This report describes the case of a previously healthy young adult non‑smoker who developed vaping product use-associated lung injury, highlighting the need for vigilance in evaluating acute respiratory illness in the context of vaping.

## Case presentation

A 22‑year‑old, previously healthy, male college student presented to the emergency department with a 7‑day history of progressive shortness of breath, non‑productive cough, pleuritic chest discomfort, intermittent low‑grade fever, and profound fatigue. He reported reduced exercise tolerance, becoming dyspneic after walking short distances -- a marked decline from his baseline.

The patient denied a history of tobacco smoking, exposure to biomass fuels, or known occupational inhalational hazards. He admitted to the frequent use of a commercially available flavored nicotine‑free vaping device for the past eight months, with daily use escalating over the preceding four weeks to multiple sessions per day. He denied use of THC or illicit substances; however, on further questioning, he acknowledged occasional purchase of refill cartridges from informal online vendors of uncertain origin approximately one month before symptom onset.

There was no history of recent travel, sick contacts, or known exposure to respiratory infections. His medical history was unremarkable, with no known allergies and no regular medications. He denied prior episodes of asthma, pneumonia, or other chronic respiratory conditions. His family history was negative for chronic lung disease or autoimmune disorders.

On examination, the patient appeared mildly distressed and tachypneic, with a respiratory rate of 26 breaths per minute. Oxygen saturation was 88% on room air, improving to 94% with supplemental oxygen via nasal cannula at 2 L/min. Temperature was 38.1 °C, heart rate 108 bpm, and blood pressure 118/74 mmHg. He was alert and oriented. Lung auscultation revealed diffuse, bilateral fine inspiratory crackles, more pronounced at the bases, with no wheezing or stridor. Cardiac examination was normal, and there was no peripheral edema, cyanosis, or clubbing. The remainder of the physical examination was unremarkable.

Initial laboratory results showed leukocytosis with a white blood cell count of 14.8 × 10⁹/L (neutrophil predominant), elevated C‑reactive protein at 85 mg/L, and erythrocyte sedimentation rate of 48 mm/hr. Arterial blood gas on supplemental oxygen revealed PaO₂ 68 mmHg, PaCO₂ 35 mmHg, and pH 7.44, consistent with hypoxemic respiratory failure. Comprehensive metabolic panel, liver function, and renal function tests were within normal limits.

Respiratory viral PCR testing, including influenza A/B, respiratory syncytial virus, and severe acute respiratory syndrome coronavirus 2 (SARS‑CoV‑2), was negative. Urinary antigens for Legionella pneumophila and Streptococcus pneumoniae were negative. HIV testing and autoimmune screening (antinuclear antibody (ANA), antineutrophil cytoplasmic antibody (ANCA)) were also negative.

**Figure 1:**
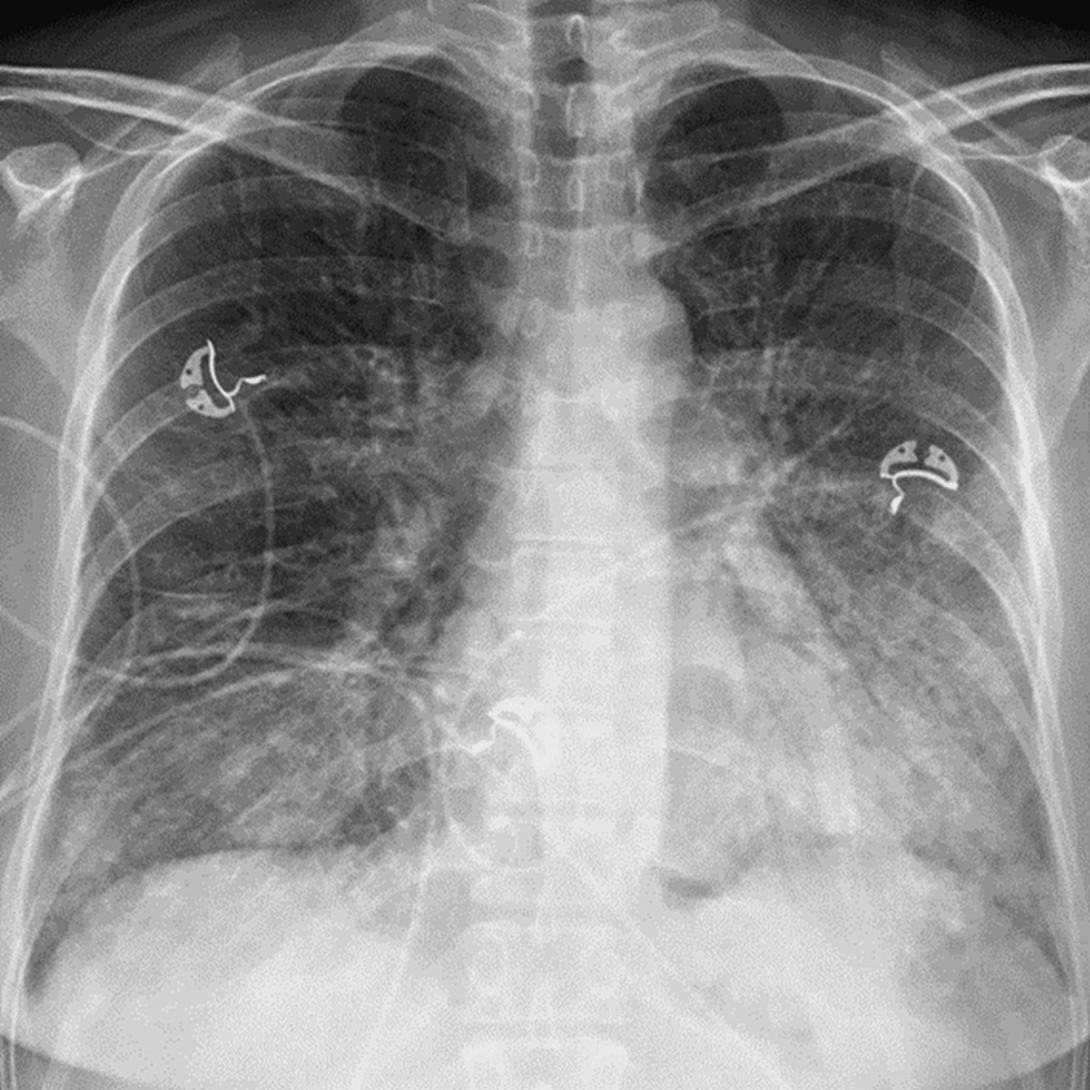
Chest radiograph demonstrating diffuse, bilateral patchy airspace opacities without lobar consolidation or pleural effusion

**Figure 2:**
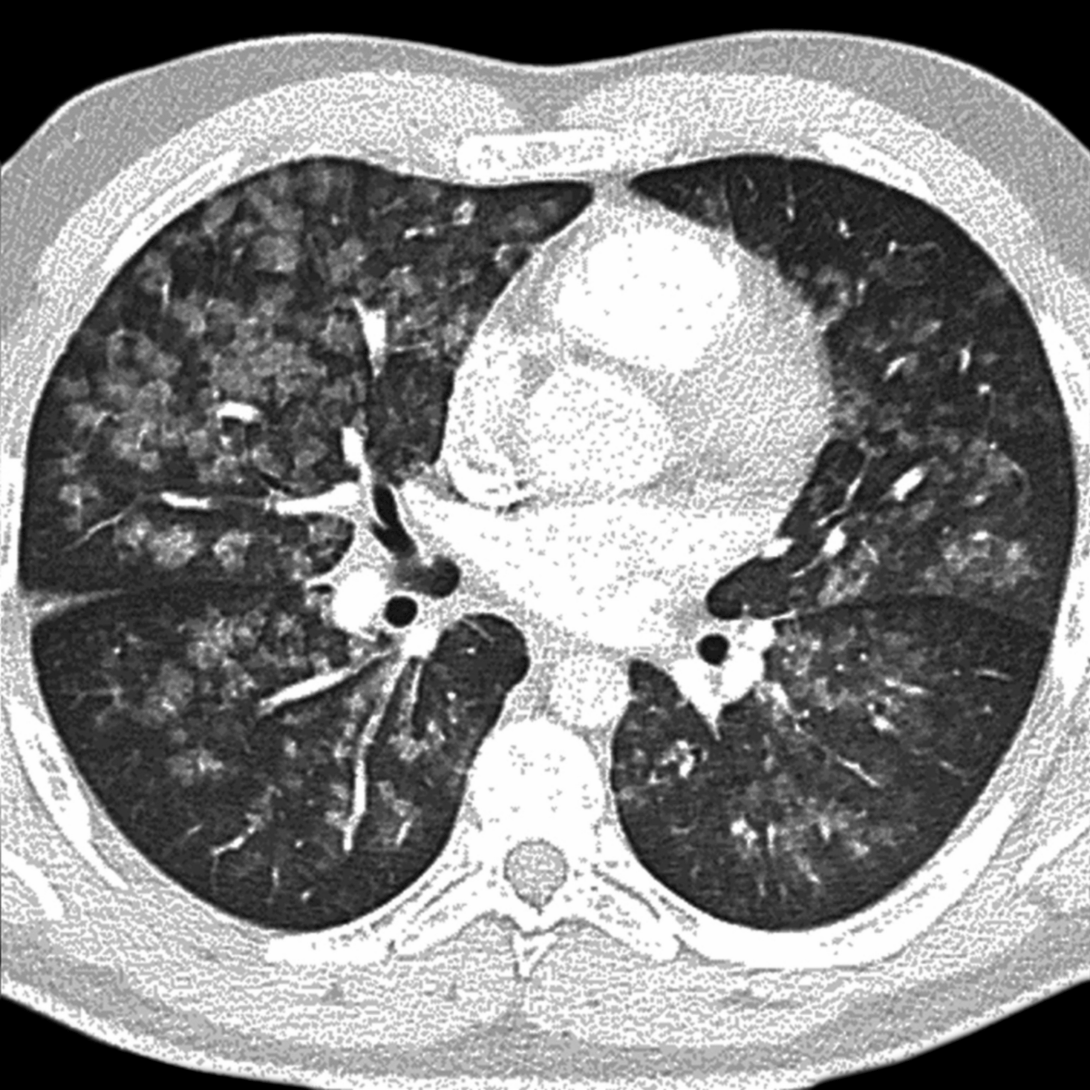
High‑resolution computed tomography of the chest showing bilateral ground‑glass opacities with areas of consolidation, predominantly in the dependent lung zones, with relative subpleural sparing No evidence of pulmonary embolism or bronchiectasis is present.

Chest radiography showed diffuse, bilateral patchy airspace opacities without lobar consolidation or pleural effusion (Figure 1). High‑resolution computed tomography revealed bilateral ground‑glass opacities with areas of consolidation, predominantly in the dependent lung zones, with relative subpleural sparing. There was no evidence of pulmonary embolism or bronchiectasis (Figure 2).

Given the imaging findings and strong temporal association with recent intensive vaping, the differential diagnosis included community‑acquired atypical pneumonia, viral pneumonitis, acute eosinophilic pneumonia, organizing pneumonia, hypersensitivity pneumonitis, and diffuse alveolar damage secondary to inhalational exposure.

In the absence of identifiable infectious pathogens, a presumptive diagnosis of vaping product use-associated lung injury was made. Empiric intravenous ceftriaxone and azithromycin were started pending culture results, along with supportive oxygen therapy. Intravenous methylprednisolone 60 mg daily was initiated after excluding active infection.

Over the next 72 hours, the patient improved markedly, with decreased oxygen requirement and resolution of fever. Antibiotics were discontinued after five days when all cultures remained negative. He was transitioned to an oral prednisone taper over three weeks. The patient was counseled extensively about the risks of vaping and advised to discontinue all e‑cigarette and vaping products. Social work and addiction counseling were arranged to support cessation.

He was discharged on hospital day seven with oxygen saturation of 96% on room air at rest and only mild exertional dyspnea. At the two‑week follow‑up, he reported complete symptom resolution and had a normal physical examination. Repeat chest radiography showed near‑complete resolution of infiltrates. At three months, pulmonary function testing revealed normal spirometry and diffusion capacity. He remained abstinent from vaping and had no recurrence of respiratory symptoms.

## Discussion

Vaping product use-associated lung injury is a recently recognized but potentially severe respiratory illness. It was first described during a 2019 outbreak in the United States. While early investigations implicated THC‑containing cartridges adulterated with vitamin E acetate, subsequent reports have shown that this injury can occur with nicotine‑only or nicotine‑free products, suggesting additional harmful agents may be involved [2-4].

The pathophysiology is incompletely understood but likely involves a combination of direct chemical toxicity, oxidative stress, and immune‑mediated inflammatory injury. Potentially harmful components include aerosolized solvents such as propylene glycol and vegetable glycerin, aldehyde flavoring agents, heavy metals from heating coils, and lipid‑containing diluents [5,6]. Some lipophilic substances may cause exogenous lipoid pneumonia, while high‑temperature vaporization can generate reactive carbonyl species capable of damaging the alveolar-capillary membrane. Histopathological findings vary and may include organizing pneumonia, diffuse alveolar damage, acute fibrinous pneumonitis, or bronchiolitis [4-6].

The diagnosis can be challenging because symptoms overlap with many other respiratory illnesses, including bacterial or viral pneumonia, acute eosinophilic pneumonia, hypersensitivity pneumonitis, and autoimmune interstitial lung disease. The Centers for Disease Control and Prevention (CDC) case definition requires a history of vaping within the previous 90 days, compatible imaging findings, and no alternative diagnosis [1-3]. Our patient met these criteria after an extensive negative infectious work‑up. His high-resolution computed tomography (HRCT) pattern of bilateral ground‑glass opacities with dependent predominance and subpleural sparing is typical of this condition and likely reflects inflammatory exudation rather than fibrosis.

Management is mainly supportive. Corticosteroids are commonly used and often lead to rapid clinical improvement, as in our case. However, they should be initiated only after excluding active infection, as they may worsen unrecognized infections. The long‑term outcomes remain uncertain; some patients recover fully, while others have persistent diffusion impairment or radiographic changes months after the acute episode. Follow‑up with pulmonary function testing and imaging is advisable [5-8].

This case adds to evidence that vaping product use-associated lung injury can occur in individuals with no history of tobacco or THC use, broadening the recognized at‑risk population. It highlights the need for healthcare providers to take detailed vaping histories, including device type, product composition, and source of cartridges, even in patients who identify as non‑smokers. Unregulated online and informal markets pose particular risks, as chemical composition is often unknown.

From a public health perspective, this case challenges the perception that vaping products labeled as “nicotine‑free” or “safer than cigarettes” are without harm. Ongoing surveillance, chemical analysis of vaping aerosols, and stricter product regulation are essential. Clinicians should maintain a high index of suspicion for this condition in patients presenting with acute respiratory symptoms and a recent vaping history, regardless of the stated nicotine or THC content [1,5].

## Conclusions

This case illustrates that vaping product use-associated lung injury can occur in previously healthy, non‑smoking young adults using nicotine‑free vaping products. The patient’s rapid improvement following corticosteroid therapy, together with the exclusion of infectious causes, underscores the importance of considering this condition early in the differential diagnosis of acute hypoxemic respiratory illness with compatible imaging.

Given the variable chemical composition of vaping products, especially those obtained from unregulated sources, clinicians should obtain a detailed vaping history in all patients with unexplained respiratory symptoms. Public health strategies must continue to address vaping risks through stronger regulation, product safety evaluation, and public education. Continued research is needed to clarify the mechanisms of injury, identify causative agents beyond vitamin E acetate, and establish standardized diagnostic and treatment guidelines to improve patient outcomes.
